# Supplementary figures and images for: Association of Serum Levels of Plasticizers Compounds, Phthalates and Bisphenols, in Patients and Survivors of Breast Cancer: A Real Connection?
Source: Int J Environ Res Public Health. 2022 Jun 30;19(13):8040. doi: 10.3390/ijerph19138040 (PMC9265398; doi:10.3390/ijerph19138040)

**Figure S1: Flow chart of participants recruitment**

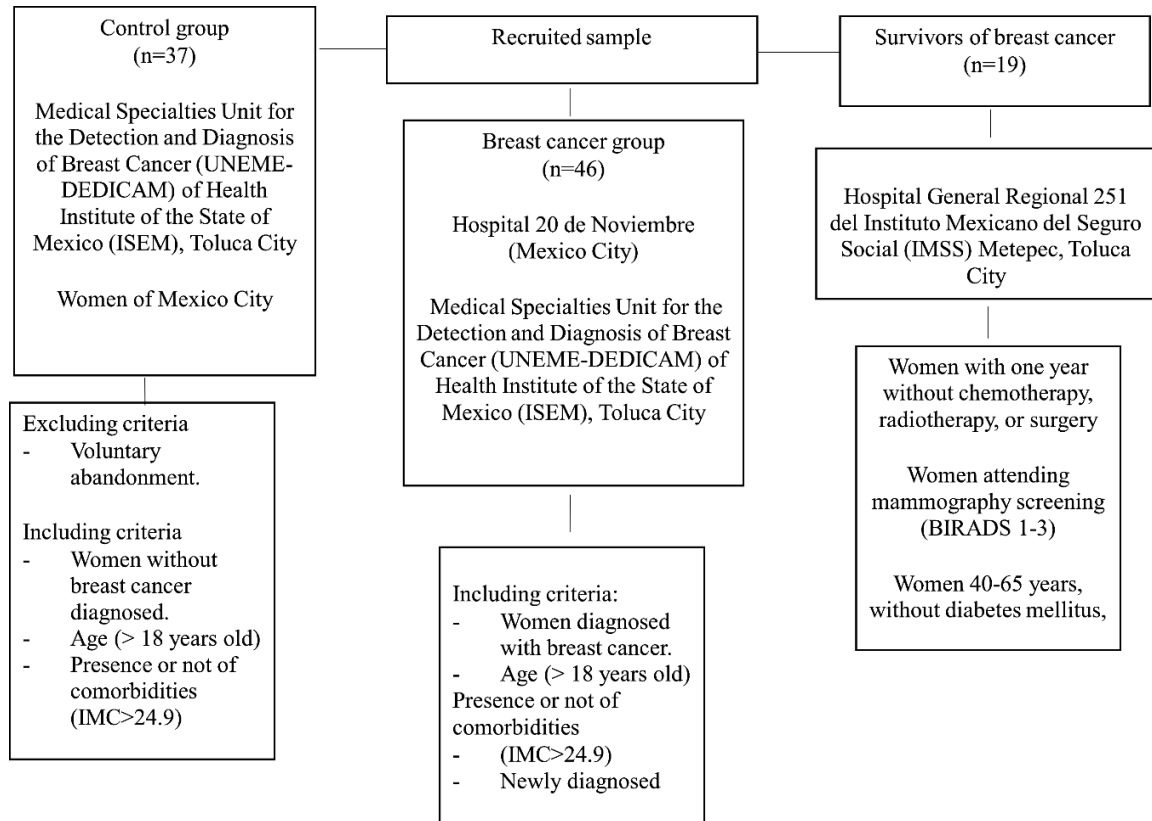

Supplement: Supplementary file 1 [file ijerph-19-08040-s001.zip › ijerph-1700839-supplementary.pdf]
